# Disruption of the Putative Vascular Leak Peptide Sequence in the Stabilized Ricin Vaccine Candidate RTA1-33/44-198

**DOI:** 10.3390/toxins5020224

**Published:** 2013-01-30

**Authors:** Laszlo Janosi, Jaimee R. Compton, Patricia M. Legler, Keith E. Steele, Jon M. Davis, Gary R. Matyas, Charles B. Millard

**Affiliations:** 1 Walter Reed Army Institute of Research, Silver Spring, MD 20910, USA; E-Mails: lacipaci@yahoo.com (L.J.); steeleK@medImmune.com (K.E.S.); gmatyas@hivresearch.org (G.R.M.); 2 NOVA Research, Inc., Alexandria, VA 22308, USA; E-Mail: jaimee.compton.ctr@nrl.navy.mil; 3 Naval Research Laboratories, 4555 Overlook Ave., Washington, DC 20375, USA; 4 United States Army Medical Research Institute of Infectious Diseases, Frederick, MD 21702, USA; E-Mail: jon.davis@osd.mil; 5 U.S. Army Medical Research and Materiel Command, Fort Detrick, MD 21702-5012, USA; E-Mail: charles.b.millard@us.army.mil

**Keywords:** ricin, recombinant vaccines, vascular leak peptide, amino acid substitutions, BALB/c mice, D75N/R48C/T77C, RTA1-33/44-198

## Abstract

Vitetta and colleagues identified and characterized a putative vascular leak peptide (VLP) consensus sequence in recombinant ricin toxin A-chain (RTA) that contributed to dose-limiting human toxicity when RTA was administered intravenously in large quantities during chemotherapy. We disrupted this potentially toxic site within the more stable RTA1-33/44-198 vaccine immunogen and determined the impact of these mutations on protein stability, structure and protective immunogenicity using an experimental intranasal ricin challenge model in BALB/c mice to determine if the mutations were compatible. Single amino acid substitutions at the positions corresponding with RTA D75 (to A, or N) and V76 (to I, or M) had minor effects on the apparent protein melting temperature of RTA1-33/44-198 but all four variants retained greater apparent stability than the parent RTA. Moreover, each VLP(−) variant tested provided protection comparable with that of RTA1-33/44-198 against supralethal intranasal ricin challenge as judged by animal survival and several biomarkers. To understand better how VLP substitutions and mutations near the VLP site impact epitope structure, we introduced a previously described thermal stabilizing disulfide bond (R48C/T77C) along with the D75N or V76I substitutions in RTA1-33/44-198. The D75N mutation was compatible with the adjacent stabilizing R48C/T77C disulfide bond and the *T*_m_ was unaffected, whereas the V76I mutation was less compatible with the adjacent disulfide bond involving C77. A crystal structure of the RTA1-33/44-198 R48C/T77C/D75N variant showed that the structural integrity of the immunogen was largely conserved and that a stable immunogen could be produced from *E. coli*. We conclude that it is feasible to disrupt the VLP site in RTA1-33/44-198 with little or no impact on apparent protein stability or protective efficacy in mice and such variants can be stabilized further by introduction of a disulfide bond.

## Abbreviations

BALF: bronchoalveolar lavage fluid
CD: circular dichroism
DTNB: 5,5'-dithiobis(2-nitrobenzoic acid)
i.m.: intramuscular
i.p.: intraperitoneal
MES: 2-(*N*-morpholino)ethanesulfonic acid
PBS: phosphate buffered saline
PEG: polyethylene glycol
RIP: ribosome inactivating protein
rmsd: root-mean-square deviation
RiVax: ricin A-chain variant containing the V76M and Y80A mutations
RTA: ricin toxin A-chain
RTA1-33/44-198: ricin toxin A-chain containing residues 1–198 and a deletion of residues 34–43, also known as RVEc
RTB: ricin toxin B-chain
SDS-PAGE: sodium dodecyl sulfate polyacrylamide gel electrophoresis
*T*_m_: melting temperature
TTD: time to death
TTDD: time to disease score drop
TTWG: time to body weight gain
VLP: vascular leak peptide
VLS: vascular leak syndrome

## 1. Introduction

Ricin is a highly toxic ribosome inactivating protein (RIP) and a potential bioterrorist and warfare agent that can be obtained easily from the widely available castor bean, *Ricinus communis* [1,2,3]. There is no known antidote but toxoid and recombinant vaccines have been shown to be effective in raising protective immunity that can prevent the lethal effects of ricin [4]. 

RiVax, a recombinant immunogen based upon the ricin toxin A-chain (RTA) with substitutions at V76M and Y80A, protects laboratory animals against ricin toxicity [5,6,7] and is safe and immunogenic for humans in Phase 1 clinical trials [8]. Tyr-80 is an active-site residue important to the catalytic mechanism of RTA as a RIP (Figure S1), whereas V76 is part of a vascular leak peptide (VLP) motif that may cause a dangerous vascular leak syndrome (VLS) in humans [5,7,9,10]. Preliminary data suggest that VLS is promoted by binding of an exposed X1-D-X2 motif present in the structure of several immunotoxins, interleukin-2, *Pseudomonas* exotoxin A fragments, or ricin with unidentified receptor(s) on target human endothelial cells (see [9]). The quantities of VLP required to elicit a human response are unknown but the V76M substitution is hypothesized to improve the overall safety profile for RiVax by disrupting the VLP in RTA comprising residues ^74^L^75^D^76^V [11]. 

A second, recombinant, RTA-based immunogen, RTA1-33/44-198 (also called RVEc), protects non-human primates from ricin and is currently under assessment in human clinical trials [12]. RTA1-33/44-198 was derived from RTA by introducing extensive deletions to reduce enzymatic activity and to enhance the structural stability of the molecule [13,14]. RTA1-33/44-198 has a reduced tendency to self-aggregate in solution compared with RTA, which may significantly reduce the costs of producing or stockpiling the vaccine to protect personnel who are at risk of exposure. However, RTA1-33/44-198 still retains the VLP and the active-site residue, Tyr-80. No enzymatic RIP activity or toxicity has been observed for the RTA1-33/44-198 A-chain truncation [13] and this is most likely due to the loss of a significant proportion of the substrate binding site and membrane binding B-chain (Figure S1). One unintended consequence of these truncations, however, may be a change in exposure and/or accessibility of the VLP in the RTA1-33/44-198 variant when compared to RTA (Figure S1, Panels D and E). The significant *C*-terminal truncation renders RTA1-33/44-198 sufficiently different in estimated protein surface area and overall compactness compared with RTA [15] to raise questions concerning the functional role of the VLP on this new scaffold; RTA1-33/44-198 could have increased or decreased affinity for VLP receptors(s) compared to RTA. The uncertainty is compounded by the relative paucity of data on human VLS, lack of accepted animal models for human VLS, and the unclear nature of specific binding interactions of the VLP with target cells *in vivo.*

A third class of potential RTA-based immunogens includes disulfide-bond stabilized variants of RTA1-33/44-198 with enhanced apparent thermal stability, including R48C/T77C and V49C/E99C [15]. The disulfide bond was found to restrict a relatively disordered loop between residues 44–55 and enabled crystallization of two variants, R48C/T77C (PDB 3LC9) and V49C/E99C (3MK9) [15]. The crystal structures, PDB 3LC9 and 3MK9, show that only a minor change in a T-cell epitope, conversion of a helix-turn-helix to a helical segment, occurs due to the truncation of the *C*-terminal 199–267 residues. The B-cell epitopes recognized by two different neutralizing antibodies remain apparently unchanged and could be superposed onto RTA. The T77C mutation is adjacent to the RTA VLP and, therefore, it was unclear whether mutations would be accommodated in the RTA1-33/44-198 disulfide-bonded variants without compromising protein stability. 

While VLS has not been reported to occur from the relatively small quantities of RTA immunogen required for vaccination, there may be concerns of adverse reactions arising from a locally high concentration at the site of injection of a protein containing the VLP. In the present study, our goal is to refine RTA1-33/44-198 further by disrupting the putative VLP by identifying compatible mutations with RTA1-33/44-198 and the disulfide-bonded variants. We developed and applied an intranasal ricin challenge model in BALB/c mice and characterized several new derivatives of RTA1-33/44-198. Our findings suggest that it is feasible to harmonize the RiVax and RTA1-33/44-198 concepts to arrive at an immunogen with enhanced stability that lacks the VLP motif. 

## 2. Results

### 2.1. Determination of the LD50 of Ricin in BALB/c Mice in the Intranasal Challenge Model

**Figure 1:**
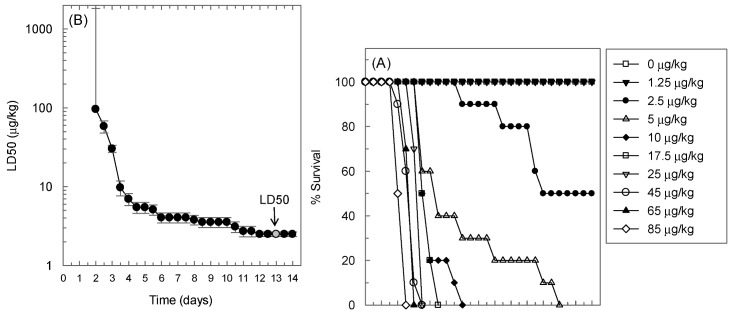
Ricin LD50 in the intranasal challenge model of BALB/c mice. (**A**) Dose- and time-dependence of survival. Ten female BALB/c mice were challenged with the indicated amounts of ricin and observed twice per day for a period of two weeks; (**B**) Time- dependence of calculated LD50. The data shown in Panel A were submitted to probit analysis to determine the LD50 of ricin at every 0.5-day intervals following ricin challenge and the calculated LD50 values were plotted versus time. The expression “LD50” with an arrow indicates that we regarded a challenge with 2.5 μg/kg ricin with a 13 day post-challenge observation window as a representative LD50 in this model.

We previously characterized an intranasal challenge model in which ricin is administered in a drop-wise manner from a micro-pipettor onto the nostrils of anesthetized mice for inhalation [16]. To determine the LD50 in this model, groups of ten 14–16 week old female BALB/c mice were challenged with varying amounts of ricin and the animals were observed twice daily for survival for 14 days. The survival curves in Figure 1. Panel A shows a clear inverse relationship between the applied ricin dose and percent survival. Below 2.5 μg/kg dose, ricin was not lethal in this model. There was a 1.5-day lag period between ricin administration and the first fatality even with the highest dose applied. Figure 1 Panel B shows that the calculated ricin LD50 depended on the duration of observation. This dependence was more apparent with shorter observation periods. Our results demonstrate that the calculated LD50 reached a plateau in the second week of the observation period and showed the greatest stability between day 12 and 14 post-challenge. We estimate that 2.5 ± 0.2 μg/kg is the LD50 of ricin in this model because of the relative stability of the LD50 value during this period. The LD50 in our intranasal challenge model is close to the LD50 values found by other laboratories for mice exposed to ricin via the airways of between 4 and 12 μg/kg depending on the actual challenge model and mouse strain used [7,17,18].

**Figure 2:**
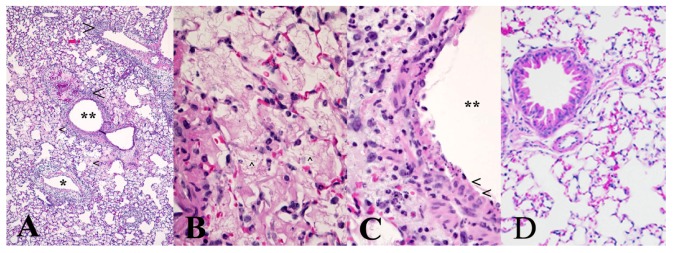
Mouse lung histopathology after intranasal ricin challenge. Ten female BALB/c mice were challenged intranasally with 10 μg/kg ricin, and on day 2, post-challenge mice were anesthetized and their lungs processed for histopathology. The panels above are representative photographs of the pathological changes in mice exposed to ricin. (**A**) Inflammatory cell infiltrates (large arrowheads) surround bronchi (**) and blood vessels (*), and many alveoli are filled with proteinaceous fluid (edema) evident as pink staining material (small arrowheads). Note also the presence of perivascular edema and hemorrhage affecting the vessel adjacent to the small bronchus; (**B**) Alveoli are largely filled by fibrin (arrowhead), and there are also some degenerate neutrophils and necrotic debris evident; (**C**) Only a limited region of the epithelium lining a bronchus remains viable (arrowheads); the remainder is necrotic. Note the edema of the peribronchial connective tissue and infiltration by neutrophils, many of which are degenerate. The pathological changes evident can be compared to the normal mouse lung histology shown in (**D**).

We carried out a limited histopathology study on the lungs of mice challenged intranasally with 10 μg/kg ricin to determine if the administered ricin reached the alveoli in this model. As shown in Figure 2, the pathological changes two days after challenge were patchy and characteristic of an acute inflammatory response similar to the changes observed in other inhalational models of ricin challenge [2,7,17]. Thus, the presence of proteinaceous material in the alveoli, perivascular and peribronchial edema, as well as cellular infiltration mostly composed of neutrophils into the lung tissue were all obvious and indicative of damage caused by ricin.

### 2.2. Protective Immunity Obtained with RTA1-33/44-198

Three groups of twenty 6 to 8 week old female BALB/c mice were immunized intramuscularly (i.m.) with 2.5 μg, 10 μg, or 40 μg/mouse of RTA1-33/44-198. The immunogen was administered in the presence of Rehydrogel adjuvant. To obtain non-immune control mice, a group of 20 mice received adjuvant only (sham vaccinated). Booster immunizations two and four weeks later were carried out with the same doses. Four weeks after the last booster, when the age of the mice became identical with the age of the mice used in the LD50 determination, the mice were challenged intranasally with 10 LD50 ricin. In addition, one group of mice that was vaccinated with 10 μg doses of the immunogen received PBS (the solvent used for the dilution of ricin throughout the experiments) in place of ricin (sham challenged) to obtain background information on the challenge. We observed the animals for survival, disease signs and body weights. Two days after challenge, 10 mice in each group were sacrificed to determine the concentration of protein in the bronchoalveolar lavage fluid (BALF) and blood glucose concentration. Observation of survival, disease signs and body weight was continued with the rest of the mice until day 13 post-challenge when they were sacrificed and BALF protein and blood glucose concentrations were determined. The data were evaluated in two ways. First, the data of the 2.5 μg, the 10 μg and the 40 μg/mouse vaccine groups were compared to the data from the sham vaccinated and the sham challenged groups. This allowed us to give a more detailed characterization of the protective effect obtained with RTA1-33/44-198 than was made before. Second, we compared the data of the 2.5 μg and the 40 μg vaccine groups to the data of the 10 μg vaccine group to see which of the above endpoints discriminated among the different vaccine doses and would be useful for comparison of the protection obtained with RTA1-33/44-198 and its VLP mutants.

**Figure 3:**
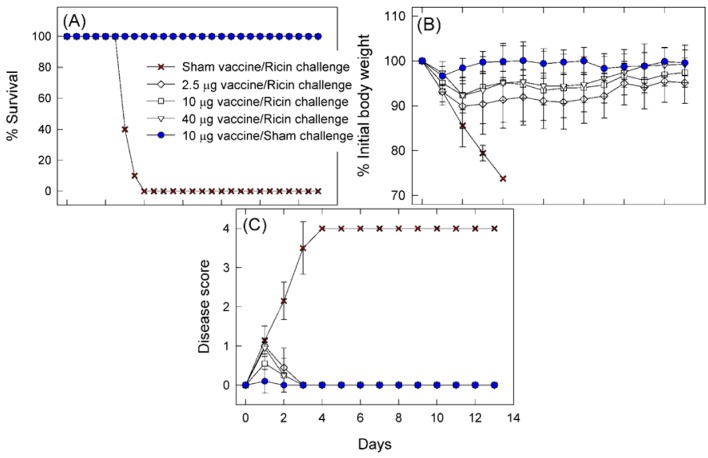
Protection provided by different doses of RTA1-33/44-198 vaccine against intranasal challenge with 10 LD50 ricin. Groups of 20 mice were vaccinated i.m., either with 2.5 μg, 10 μg, or 40 μg/mouse RTA1-33/44-198, in the presence of Rehydrogel adjuvant with two booster immunizations two weeks apart after priming. One group of mice received only the adjuvant (sham vaccine). Four weeks after the last booster immunization, the mice were intranasally challenged either with 10 LD50 ricin in PBS or PBS lacking ricin (sham challenge). Mice were observed twice daily for survival (**A**), once daily for body weights (**B**) and once daily for disease signs (**C**). Qualitative grading of the disease between 0 and 4 was as described in Materials and Methods. In panels (**A**) and (**B**), symbols represent group mean values and vertical bars show one standard deviation.

In Figure 3 Panel A we show that all mice immunized with 2.5, 10 or 40 μg/mouse doses of RTA1-33/44-198 in the presence of adjuvant were completely protected from the lethal effect of ricin in this challenge model. In contrast, the mean time-to-death value associated with the mice vaccinated with adjuvant only (sham vaccine) was 3.2 ± 0.1 days. The results obtained with mice that had been vaccinated with 10 μg doses confirmed earlier findings with this immunogen obtained in intraperitoneal and whole body ricin exposure models after challenges with the same amount of ricin [13]. We also investigated the impact of altering the dose of immunogen in our challenge model. We found that neither a 4-fold decrease nor increase in the amount of the immunogen (to 2.5 μg per dose and to 40 μg per dose, respectively) had an impact on survival. In all cases, the vaccine was 100% effective in preventing death.

The daily body weight data collection in the 13-day observation window allowed us to make a quantitative analysis of the dose dependent protective effects of the immunizations. All vaccine doses (2.5, 10 or 40 μg immunogen) resulted in significant protection from ricin-induced weight loss when compared to unvaccinated animals. The data shown in Panel B of Figure 3 allowed us to analyze both the time aspects and the magnitude of these body weight changes. Thus, the mean time to weight gain (TTWG) of the 2.5 μg vaccine dose group (3.6 ± 0.4 days), the 10 μg vaccine dose group (3.4 ± 0.2 days) and the 40 μg vaccine dose group (3.5 ± 0.3 days) were all significantly improved compared with the >13 days required for sham vaccinated mice exposed to ricin. There was no dose-dependent impact of vaccination on the weight gain of the animals, although vaccinated animals challenged with ricin exhibited significantly longer TTWG compared to controls (Figure 3B)**.** Even though we did not find a dose-dependent effect on TTWG, the animals vaccinated with 2.5 μg demonstrated significantly less total weight gain when compared to the 10 μg and 40 μg doses. All animals challenged with ricin, with the exception of the 40 μg group, demonstrated significantly reduced weight at the end of the observation period compared to controls; the 40 μg group had weights comparable to the controls (Figure 3). 

Qualitative signs of ricin-induced toxicity (diseases scores) progressively increased for immune naïve (sham vaccine) animals until they died (Figure 3C). In all other groups, there was an increase in disease score following the challenge that did not exceed a score of 1 and disease score returned to 0 by day 3 post-challenge. The statistical analysis showed that the 0.2 ± 0.2 day mean time to disease score drop (TTDD) associated with the sham challenge group was significantly shorter than the 2.4 ± 0.1 (*p* < 0.001), the 1.3 ± 0.3 (*p* = 0.005) and the 2.1 ± 0.1 (*p* < 0.001) days TTDD in the 2.5, 10 and 40 μg vaccine dose groups, respectively. Interestingly the TTDD was shorter with the 10 μg dose group than in both the 2.5 and the 40 μg vaccine dose group. The differences were significant, perhaps indicating underlying pathological process associated with immune reactions. On day 2 post-challenge, immediately before sacrifice, the group disease scores recorded of 0.5 ± 0.5 (2.5 μg dose group) or 0.2 ± 0.4 (both the 10 μg and the 40 μg dose groups) were significantly different from the sham challenge groups (*p* = 0.001, and *p* = 0.019, respectively). We did not observe a dose dependent protective effect for disease score, probably because of the low scores and the observer dependent nature of this test. The qualitative disease scores suggested that the vaccine does not completely protect against all signs of intoxication during the early stages following ricin exposure but that all measured signs do resolve within a short period of time; this is consistent with the quantitative changes in post-challenge animal body weight.

**Figure 4:**
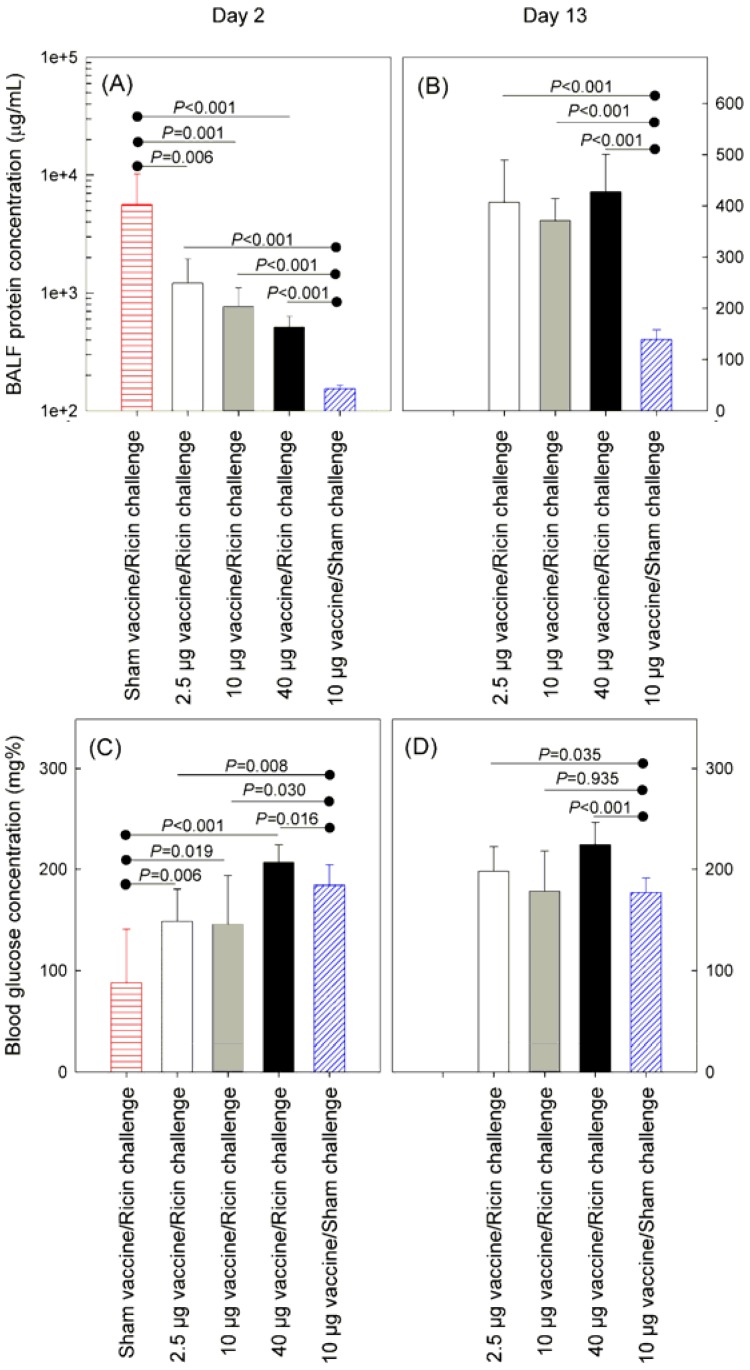
Protection provided by different doses of RTA1-33/44-198 vaccine against intranasal challenge with 10 LD50 ricin. Groups of 20 mice were vaccinated i.m., either 2.5 μg, 10 μg, or 40 μg/mouse RTA1-33/44-198, in a prime-booster-booster strategy two weeks apart. One group of mice received only the adjuvant, but not the immunogen (sham vaccine). Four weeks after the last booster immunization, the mice were challenged intranasally either with 10 LD50 ricin in PBS or PBS lacking ricin (sham challenge). On day 2 post-challenge, 10 mice in each group were sacrificed, and protein concentration in the BALF and total blood glucose concentrations were determined. The protein and glucose concentrations were repeated on day 13. (**A**) Protein concentrations in the BALF on day 2 post-challenge; (**B**) Protein concentrations in the BALF on day 13 post-challenge; (**C**) Blood glucose concentrations on day 2 post-challenge; (**D**) Blood glucose concentrations on day 13 post-challenge. In all panels, the columns represent group mean values, and the vertical bars show one standard deviation. *p* values above the lines with a knob on the left were calculated using an unpaired *t*-test to compare group mean values between the sham vaccine group and each group of vaccinated animals following ricin exposure; *p* values above the lines with a knob on the right were calculated to compare group mean values between the sham challenge group with each group of vaccinated animals.

The BALF protein content following ricin challenge is a marker of the pathological process elicited by the toxin in the lungs [2,17]. Recently another marker, an acute decrease in the blood glucose concentration, was described [19]; the decrease may indicate the deleterious effect of ricin on the metabolic processes of the organism. In this study, we examined both BALF protein and blood glucose concentrations to further discriminate the effects of vaccination. The results of this analysis are summarized in Figure 4. Two days after challenge, the BALF protein concentration was significantly higher in the RTA1-33/44-198-vaccinated and ricin challenged animals compared to mice not exposed to ricin (Figure 4, Panel A). When compared to the immune naïve animals, all values were significantly lower in the immunized animals. Protein concentrations decreased by day 13 in all groups that received vaccine (Figure 4, Panel B); however, these concentrations were still significantly higher than in the control group. Data collected on day 2 post-challenge indicated that blood glucose concentrations decreased in ricin challenged animals (Figure 4, Panel C). All blood glucose levels were significantly higher in the RTA1-33/44-198-immunized animals on this day suggesting that blood glucose levels may serve as a biomarker of protection. 

**Table 1:** Summary of significant biomarker differences which occurred over the 13 day observation period after the 10 LD50 intranasal ricin challenge in BALB/c mice vaccinated with different doses of RTA1-33/44-198^a^.

Post-challenge time point	Vaccine dose (μg/mouse)	Significant deviation of group mean values from the values of the 10 μg vaccine dose group ^b^			
		BALF protein concentration (μg/mL)	Blood glucose concentration (mg%)	% Initial body weight (g)	Disease scores (points)
Day 1	2.5	NT	NT	Down by 1.8 (*p* = 0.032)	Up by 0.4 (*p* < 0.001)
	40	NT	NT	Up by 2.3 (*p* = 0.003)	Up by 0.4 (*p* = 0.004)
Day 2	2.5	-	-	-	-
	40	Down by 253 (*p* = 0.043)	Up by 61 (*p* = 0.001)	-	-
Day 3–12	2.5	NT	NT	-	-
	40	NT	NT	-	-
Day 13	2.5	-	-	-	-
	40	-	Up by 45 (*p* = 0.001)	-	-

^a^ Groups of 20 mice were immunized intramuscularly with varying amounts of RTA1-33/44-198 in a priming-booster-booster schedule two weeks apart. The immunogen was administered with Rehydrogel adjuvant as described in Materials and Methods. Four weeks after the last booster immunization, the mice were challenged with 10 times the calculated LD50 of ricin. Body weights and disease severity scores were determined daily throughout a 13-day observation period. On day 2 and 13 post-challenge, animals were sacrificed to determine BALF protein concentrations and total blood glucose levels. Only group mean values that significantly differed from that of the 10 μg vaccine reference group are shown (source data values are shown in Figure 3, Figure 4); ^b^ “NT” stands for not tested. “-” stands for group mean values not significantly different from the values observed in the 10 μg vaccine dose group.

Next, we wanted to determine if body weight, disease score, BALF protein concentration and blood glucose levels could discriminate among the different dosing groups. The results are summarized in Table 1. According to this analysis on day 1 post-challenge the mice vaccinated with 2.5 μg vaccine doses showed significantly (*p* = 0.032) lower body weights (93% ± 3% of the initial) than the mice vaccinated with 10 μg doses (95% ± 2%). The significance of this difference disappeared from day 2 post-challenge. The body weight of the group vaccinated with 40 μg vaccine doses (97% ± 3%) was significantly (*p* = 0.003) higher than the group of mice vaccinated with 10 μg doses. This significant difference also lasted only for 1 day post-challenge. On day 2 post-challenge, a significantly lower BALF protein concentration (*p* = 0.043) and a significantly higher blood glucose concentration (*p* = 0.001) was associated with the 40 μg vaccine group. The blood glucose concentration values in this group were still significantly higher (224 ± 22 mg%, *p* = 0.005) on day 13 post-challenge when compared to the 10 μg vaccine dose group (179 ± 40 mg%). Based on our observations, we kept body weight, BALF protein and blood glucose analyses as endpoints for a later comparison of the protection provided by RTA1-33/44-198 and its VLP mutants as described later in the text. We also used these endpoints to analyze the effects of intranasal challenges with RTA1-33/44-198 vaccine immunogen in place of ricin.

### 2.3. Intranasal Challenge with Immunogen in Place of Ricin

RTA1-33/44-198 still possesses the major murine B-cell epitopes and is immunogenic in mice [13]. Furthermore, the ^74^L^75^D^76^V tri-peptide, implicated in VLS [9,11] is present in RTA1-33/44-198, as are active site residues. The exposure of the VLP also differs between RTA (or RiVax) and RTA1-33/44-198 R48C/T77C (Figure S1D,E). We reasoned that if intranasal challenge of mice is carried out with RTA1-33/44-198 in place of ricin, we might see alteration of some of the biomarkers either as a sign of the local immune reaction in an immunized animal background, or the presence of residual toxicity of the molecule. The latter, if strong enough, might alter our selected biomarkers even in a non-immune background. The results of our experiments are shown in Table 2. On day 2 post-challenge the BALF protein and blood concentrations were significantly increased in the RTA1-33-44-198-Immune animals when compared to PBS-challenged animals. In addition, the body weights of the animals in the same group were significantly lower on day 1 post-challenge. These changes were related to the immune status of the animals, because the biomarkers were also significantly different from the values shown by the RTA1-33/44-198-naïve animals.

**Table 2:** Effect of intranasal vaccine immunogen challenges on biomarkers of naïve and RTA1-33/44-198-immunized BALB/c mice.

Endpoint ^a^	Time point ^b^	PBS challenge ^c^	RTA1-33/44-198 challenge in immune naïve mice ^d^		RTA1-33/44-198 challenge in immune mice ^e^		Significance of naïve/immune difference (*p*)
		Mean ± S.D.	Mean ± S.D.	Significance of difference from PBS challenge (*p*)	Mean ± S.D.	Significance of difference from PBS challenge (*p*)	
BALF protein (μg/mL)	Day 2	154 ± 10	178 ± 37	0.212	274 ± 66	**<0.001**	**<0.001**
	Day 13	139 ± 19	106 ± 14	**<0.001**	193 ± 39	**0.005**	**<0.001**
Blood glucose (mg%)	Day 2	184 ± 20	190 ± 20	0.573	228 ± 13	**<0.001**	**<0.001**
	Day 13	178 ± 14	194 ± 14	**0.026**	218 ± 25	**<0.001**	**0.023**
% initial weight	Day 1	96.7 ± 2.1	97.2 ± 1.8	0.399	94.6 ± 3.0	**0.016**	**0.002**
	Day 2	98.4 ± 2.2	100.1 ± 2.6	**0.038**	98.7 ± 3.0	0.746	0.137
	Day 13	99.6 ± 3.0	105.1 ± 3.0	**<0.001**	102.9 ± 2.6	**0.017**	0.088

^a^ BALF: bronchoalveolar lavage fluid; ^b^ Days after the intranasal RTA1-33/44-198 vaccine challenge; ^c^ Mice were vaccinated with 10 μg doses of RTA1-33/44-198 with Rehydrogel adjuvant as described in Materials and Methods. Intranasal challenge was with PBS. S.D.: one standard deviation; ^d^ Mice received sham vaccine (adjuvant only) with the same schedule as described for the PBS challenge group. Intranasal challenge was with RTA1-33/44/198 in an amount equimolar with 10 LD50 ricin. S.D.: one standard deviation; ^e^ Mice were vaccinated with 10 μg doses of RTA1-33/44-198 with Rehydrogel adjuvant as described in Materials and Methods. Intranasal challenge was with RTA1-33/44/198 vaccine immunogen in an amount equimolar with 10 LD50 ricin. S.D.: one standard deviation.

### 2.4. Vascular Leak Peptide Mutant Derivatives of RTA1-33/44-198 and Their Thermal Stabilities

**Figure 5:**
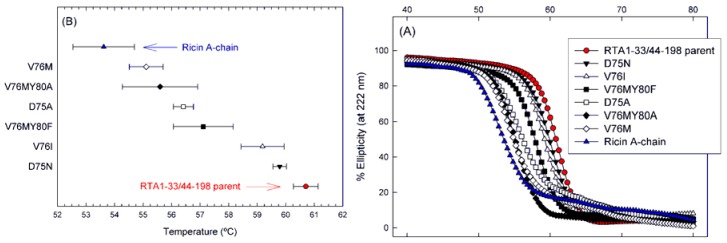
Apparent thermal stability of VLP mutants of the RTA1-33/44-198 immunogen. (**A**) Temperature melting curves. Changes in molecular structure of the proteins induced by elevated temperatures were monitored by circular dichroism (C,D) spectroscopy at 222 nm. Data are expressed in percent of the initial ellipticity observed at 10 °C; (**B**) Melting temperatures associated with 50% loss of ellipticity of the molecules. Error bars indicate one standard deviation of the data of three independent experiments.

Site-directed mutagenesis was used to introduce amino acid substitutions into the VLP [5,9,11] that were not removed by earlier sequence deletions [13] from the thermal stable RTA1-33/44-198 recombinant ricin vaccine candidate [15]. VLP substitutions D75A, D75N, V76M and V76I decreased the thermal stability of the molecule as shown in Figure 5 and Table 3 The degree of decrease varied with the amino acid introduced rather than its position. Despite the decrease in thermal stabilities, the *T*_m_ differences between RTA and the RTA1-33/44-198 VLP mutants remained significant with the exception of the RTA1-33/44-198 V76M mutant which had a *T*_m_ only 1.9 degrees higher than RTA. The RTA1-33/44-198 D75N mutant showed the smallest decrease in the *T*_m_ (1 degree), while the V76M mutant had a 5.6 degree decrease in its melting temperature (Table 3). 

We also note in Figure 5 that the shape of temperature melting curves of the mutants seem to fall into two groups. Mutants V76M and D75A were very similar to RTA and had sloped baselines possibly due to aggregation [20]. All other mutants resembled the parent RTA1-33/44-198 which, unlike RTA, shows only a single highly cooperative transition and flat baselines. This difference between RTA and RTA1-33/44-198 was discussed previously [14].

**Table 3:** Attempts to introduce a stabilizing disulfide bond at different locations in RTA1-33/44-198.

Ricin vaccine immunogen	Immunogen	Sequencing ^1^	Solubility/Purity ^2^	Number of free Cys ^3^	Apparent *T*_m_	Disruption of VLP?
Purification Method No. 1						
RTA	-	Yes	S+/>95%	-	53.5 ± 0.74	No
RTA1-33/44-198	-	Yes	S+/>95%	-	61.0 ± 0.23	No
RTA1-33/44-198	D75N	Yes	S+/>95%	-	60.0 ± 0.05	Yes
RTA1-33/44-198	V76I	Yes	S+/>95%	-	59.8 ± 0.17	Yes
RTA1-33/44-198	D75A	Yes	S+/>95%	-	56.6 ± 0.10	Yes
RTA1-33/44-198	V76M	Yes	S+/>95%	-	55.4 ± 0.42	Yes
Purification Method No. 2						
RTA1-33/44-198	-	Yes	S+/>95%	0.97 ± 0.03	57.9 ± 0.03	No
Substitutions that Increase Apparent *T*_m_						
RTA1-33/44-198	R48C/T77C	Yes	S+/>95%	1.00 ± 0.08	62.9 ± 0.22	No
RTA1-33/44-198	V49C/E99C	Yes	S+/>95%	0.96 ± 0.05	62.9 ± 0.21	No
RTA1-33/44-198	R48C/T77C/D75N	Yes	S+/>95%	1.04 ± 0.02	63.2 ± 0.26	Yes
RTA1-33/44-198	V49C/E99C/V76I	Yes	S+/>95%	0.99 ± 0.03	62.6 ± 0.21	Yes
RTA1-33/44-198	V49C/E99C/D75N	Yes	S+/>95%	1.10 ± 0.06	62.2 ± 0.13	Yes
RTA1-33/44-198	R48C/T77C/V76I	Yes	S+/>95%	1.89 ± 0.03	59.3 ± 0.60	Yes

^1^ Presence of desired substitution and no other was confirmed by DNA sequencing; ^2^ Solubility was assessed qualitatively by evaluating Coomassie blue stained SDS-PAGE gels of initial induction checks. Cells over-expressing recombinant protein were fractionated and scored as I (>50% detected in the insoluble pellet), S (>50% in soluble fraction), or S+ (entirely in soluble fraction). Purity of final product was estimated from Coomassie blue-stained SDS-PAGE. The identity of purified protein was confirmed by reaction with antisera on Western blots; ^3 ^The number of free Cys residues was quantified for purified proteins by titrating free –SH using 5,5'-dithiobis-(2-nitrobenzoic acid (DTNB). DTNB titrations were conducted in triplicate at four different protein concentrations between 2 and 11 μM; the mean ± SE was determined from linear regression of the titration plots. Note that the parent protein molecule (RTA1-33/44-198) contains one Cys, whereas each mutant contains a total of three Cys residues. The DTNB assay was optimized to probe the reduced Cys residues such that values significantly greater than one reflect incomplete formation of a stable disulfide bond between the engineered Cys residues.

### 2.5. Protective Immunity Obtained with the VLP Mutants of RTA1-33/44-198

We wanted to determine if the mutations introduced into RTA1-33/44-198 had inadvertently diminished the immunogenicity of the molecule. To achieve this, groups of mice were vaccinated under identical conditions (10 μg/mouse i.m. administered doses in a priming-booster-booster vaccination schedule) with the parent RTA1-33/44-198 vaccine and its mutant derivatives, followed by identical supralethal ricin challenge (10 LD50 intranasally administered ricin). In addition to monitoring challenged animals for overall survival, we also measured the change in our previously selected biomarkers. 

All of the vaccine immunogens fully protected mice from ricin lethality. We then compared the BALF protein concentrations, blood glucose concentration and body weight values of the animals vaccinated with the VLP mutants to the animals vaccinated with RTA1-33/44-198. The results are shown in Table 4. The only vaccine immunogen that performed slightly weaker than its parent in the day 2 post-challenge evaluation was D75A. D75A allowed significantly higher BALF protein content (group mean value up by 541 μg/mL) and lower body weights (group mean value down by 5.8 g). The BALF protein content increase was transient and disappeared by day 13 post-challenge with this VLP mutant in comparison with its parent molecule. Three VLP mutant vaccines, V76I, V76M and V76M/Y80F appeared to protect the mice better from the ricin-elicited blood glucose decrease in the day 2 post-challenge evaluation as shown in Table 4, but the other biomarkers did not differ significantly from the values shown by the parent vaccine. Finally, we could not see any significant difference between the single point mutants, D75N and V76M, and the double mutant V76M/Y80A by these tests when compared to the parent RTA1-33/44-198. 

**Table 4:** Comparison of the protective immunity provided by RTA1-33/44-198 and its VLP mutants after 10 LD50 intranasal ricin challenge in BALB/c mice^a^.

Post-challenge time point	Vaccine ^b^	Significant alteration of group mean values from the group mean values of the animals immunized with RTA1-33/44-198 ^c^		
		BALF protein concentration (μg/mL)	Blood glucose concentration (mg%)	% Initial body weight (g)
Day 1	D75A	NT	NT	-
	D75N	NT	NT	-
	V76I	NT	NT	-
	V76M	NT	NT	-
	V76M/Y80A	NT	NT	-
	V76M/Y80F	NT	NT	-
Day 2	D75A	Up by 541 (*p* = 0.001)	-	Down by 4.7 (*p* = 0.001)
	D75N	-	-	-
	V76I	-	Up by 61 (*p* = 0.002)	-
	V76M	-	Up by 45 (*p* = 0.012)	-
	V76M/Y80A	-	-	-
	V76M/Y80F	-	Up by 37 (*p* = 0.036)	-
Day 13	D75A	Down by 93 (*p* < 0.001)	-	-
	D75N	-	-	-
	V76I	-	-	-
	V76M	-	-	-
	V76M/Y80A	-	-	-
	V76M/Y80F	-	-	-

^a^ Groups of 20 mice were immunized intramuscularly either with 10 μg RTA1-33/44-198 or with the same doses of its VLP mutant in a prime-boost-boost schedule two weeks apart. The immunogens were administered together with Rehydrogel adjuvant as described in Materials and Methods. Four weeks after the last booster immunization, the mice were intranasally challenged with 10 times the LD50 ricin. Body weights were measured daily starting immediately before the challenge. Two days after the challenge, animals were sacrificed to determine the BALF protein concentrations and blood glucose levels. The BALF protein concentration and blood glucose measurements were repeated on day 13 post-challenge. Only group mean values that significantly differed from that of the RTA1-33/44-198 vaccine reference group are shown (source data values are shown in Figure 3, Figure 4); ^b^ VLP mutant derivatives of RTA1-33/44-198 are described by showing the amino acid substitution(s) in the immunogen; ^c^ “NT” stands for “not tested”. “-” stands for group mean values that showed no significant difference from the values observed with the RTA1-33/44-198 reference vaccine.

### 2.6. Effects of the D75N and V76I Mutations on the Thermal Stability of RTA1-33/44-198 R48C/T77C and RTA1-33/44-198 V49C/E99C

To determine which VLP mutations were compatible with the disulfide bond variants and to investigate their effect on structure, we incorporated the D75N and V76I mutations into the next generation RTA1-33/44-198 R48C/T77C and V49C/E99C thermostabilized variants. The D75A and V76M mutations adversely reduced the *T*_m_ by >3 °C when compared to the parent immunogen, RTA1-33/44-198, and were not transferred into the disulfide variants. The D75A mutation removes both a potential hydrogen bonding interaction and a salt bridge, while the D75N mutation retains the hydrogen bonding interaction (Figure S1). The loss of these interactions may account for the reduced stability of the D75A variant. In total, four triple mutants were constructed. The number of free thiols and apparent *T*_m_ were measured as previously described [15]. Three of the triple mutant variants (Table 3) had *T*_m_ values comparable to RTA1-33/44-198 R48C/T77C or V49C/E99C. Only one variant, RTA1-33/44-198 R48C/T77C/V76I, showed a 3.6 °C decrease in *T*_m_ upon incorporation of the V76I mutation. These RTA variants contain three cysteines; two involved in a disulfide bond and one free cysteine. Examination of the thiol titration data of the V76I variant showed that more than 1 free thiol (*n*_SH_ = 1.89 ± 0.03) was detected indicating that the disulfide bond was not effectively forming in this variant (Table 3). The V76I mutation is adjacent to T77C suggesting that the larger isoleucine side chain is incompatible with forming the disulfide bond. In contrast, the D75N mutation was compatible with both the R48C/T77C and V49C/E99C constructs and the *T*_m_ values were comparable to the parent molecules. This more conservative mutation was well tolerated.

**Figure 6:**
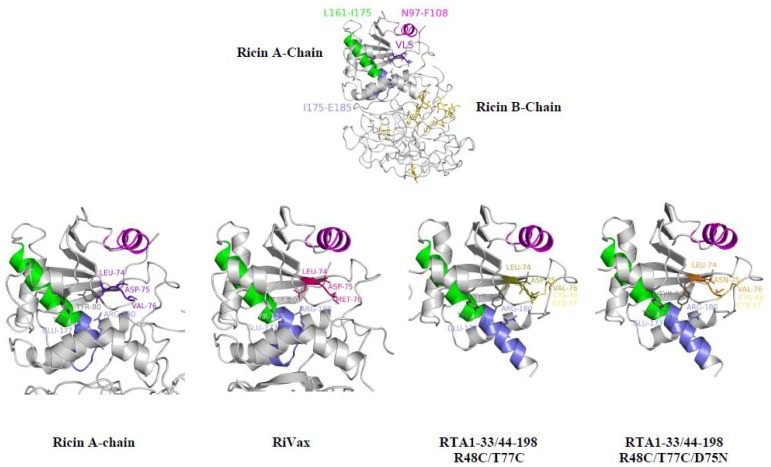
Comparison of the VLP sites among ricin and three different RTA-based immunogens (labeled in figure). The B-cell epitope recognized by the Univax R70 antibody is shown in magenta. The L161–I175 epitope bound by human neutralizing antibodies characterized by Castelletti [21] is shown in green. The T-cell epitope is shown in blue; this epitope is found to be fully helical in the truncated 1-33/44-198 disulfide-bonded immunogens.

### 2.7. Effect of the D75N Mutation on the Structure of RTA1-33/44-198 R48C/T77C

We determined the structure of the D75N mutant of RTA1-33/44-198 R48C/T77C/D75N by X-ray crystallography to evaluate the consequences of altering the VLP (data collection and refinement statistics are shown in Table 5). Suitable crystals of the RTA1-33/44-198 immunogen have not been obtained to date but the disulfide bonded variants crystallize readily; this difference may be due to the reduction in mobility of the loop containing the disulfide (residues 44–55). Crystals of the disulfide bonded variants have enabled us to examine the structural integrity of the immunogens and the effects of mutations on the RTA1-33/44-198 scaffold [15]. RTA1-33/44-198 R48C/T77C (PDB 3LC9) could be aligned with the RTA (PDB 2AAI) with an rmsd of 0.69 over 156 C_α_. Similarly, the RTA1-33/44-198 R48C/T77C/D75N (PDB 4IMV) (Table 5) aligns to RTA with a rmsd of 0.66 over 154 C_α_. The VLP sites are shown in Figure 6 and Figure S1. The thermostabilizing disulfide bond lies adjacent to the VLP. The conserved D75N mutation had no significant effect on the overall structure when compared to RTA1-33/44-198 R48C/T77C. 

**Table 5:** X-ray crystallography statistics.

RTA1-33/44-198 R48C/T77C/D65N (PDB 4IMV)	
Space group	I222
Unit Cell Dimensions (Å)	*a* = 51.6, *b* = 72.3, *c* = 94.7
Unit Cell Angles (°)	α = 90, β = 90, γ = 90
Wavelength (Å)	1.54
Resolution Range (Å)^ a^	57.5–2.25 (2.31–2.25)
Unique Reflections	8640 (860)
*R*_int_	0.073 (0.285)
I/σI	13.0 (3.6)
Completeness	99.1 (91.6)
Redundancy	6.9 (3.8)
**Refinement Statistics**	
Resolution (Å)	47.35–2.25
No. of reflections	8233
*R*_factor _^b^	0.216
*R*_free _^c^	0.242(5%)
Number of Atoms	
Protein	1318
Solvent	79
Other	4
Average B-factors (Å^2^)	
Protein	22.2
Solvent	29
R.m.s.d. from ideal geometry	
Bond lengths (Å)	0.015
Bond angles (degrees)	1.37
Ramachandran plot	
Most favored regions (%)	95.70%
Additional allowed regions (%)	4.30%
Generously allowed regions (%)	0.00%
Disallowed regions (%)	0.00%

^a ^Values in parentheses are for the outer most data shell; ^b ^*R*_factor_ for working set of reflections was calculated using: 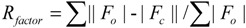 ; ^c ^*R*_free_ for test set and size of test set as % total reflections in parentheses.

## 3. Discussion

We used an intranasal challenge model in this study based on delivery of the toxin into the nostrils of anesthetized BALB/c mice [16]. Since mice are obligate nasal breathers [2] the method was expected to be an efficient way to introduce the toxin into the airways of the mice and at the same time technically less demanding than either exposure to aerosol [7,22], or intratracheal instillation [22]. Lung histopathology on a group of 10 mice provided evidence that the administered ricin, at least in part, reaches the lungs (Figure 2). The pathology showed a patchy pattern of tissue necrosis and acute inflammation characteristic of the effect of ricin on lung tissues [2,7,17]. We found that the estimated LD50 of ricin in our model is 2.5 μg/kg; this is close to the 3–5 μg/kg value observed in the aerosol and the intratracheal instillation models. The similarity between the nose-only aerosol challenge model [7] and our intranasal challenge model further extends to the time aspects of the lethal effect after a challenge of 10 LD50 ricin. The reported lag time between the exposure and the first death and the time to no survival appear to be three and four days, respectively, after aerosol challenge. These values were 2.5 and 3.5 to 4 days, respectively, in our model (Figure 1 and Figure 3). The LD50 in our model varied somewhat from the approximate 6 μg/kg value observed in the recently described and also technically simple oropharyngeal aspiration challenge model [17]. In fact, the LD50 value in the oropharyngeal model appears to be higher than the LD100 value (approximately 5 μg/kg) in our model. It is unclear if this reflects simple laboratory variation or a biologically significant difference between the two exposure routes.

Biomarkers other than survival also were altered significantly by exposure of animals to ricin in our model. In unprotected animals, we observed rapid and irreversible body weight loss and an irreversible increase in the severity of disease signs (Figure 3) similar to effects reported in other inhalational models in mice [2,7,17]. Total protein concentration increased in the BALF to significantly different levels by day 2 post-challenge (Figure 4). A similar observation was originally made in an intratracheal instillation challenge model in rats [23]. Furthermore, unprotected mice showed a decrease of the blood glucose concentration after challenge (Figure 4). This finding confirms the original finding made in CD1 mice [19] and extends it to the BALB/c strain. The mechanism of this decrease is unknown but may be associated with depletion of the glycogen depot [24].

We found that mice vaccinated with increasing doses (2.5, 10 and 40 μg) of the RTA1-33/44-198 recombinant ricin immunogen administered with Rehydrogel adjuvant i.m. were protected against a 10 × LD50 challenge with ricin. These findings are consistent with our earlier findings with this immunogen tested with >10 × LD50 ricin challenges in different challenge models, including non-human primates [13]. In addition to survival, the present work further refines our understanding of the protection conferred by this immunogen and shows its dose dependent nature. Thus, body weight losses were significantly less, disease scores and BALF protein concentrations significantly lower and blood glucose concentrations significantly higher in the vaccinated mice when compared to unprotected mice (Figure 3 and Figure 4). Furthermore, body weights were significantly lower in the mice vaccinated with 2.5 μg doses and significantly higher in mice vaccinated with 40 μg doses than in mice vaccinated with 10 μg doses of RTA1-33/44-198 (Table 1). Likewise, the BALF protein concentration was significantly lower and the blood glucose concentration significantly higher in the mice vaccinated with 40 μg doses when compared to mice vaccinated with 10 μg doses of the immunogen two days after a 10 × LD50 ricin challenge (Table 1).

Vaccination with RTA1-33/44-198 fully protected against lethality following ricin exposure in our model but we observed transient differences in several biomarkers for animals exposed to toxin compared with the values from sham-challenged (animals vaccinated with RTA1-33/44-198 and challenged with PBS lacking ricin). There were significant decreases in body weight and worsened qualitative disease scores following ricin challenge even in animals given the highest immunogen dose tested (Figure 3). Similarly, the day 2 post-challenge BALF protein concentrations were significantly elevated when compared to PBS challenge (Figure 4). The blood glucose concentrations of toxin-exposed animals also were different than those measured in the controls (Figure 4). The decreased body weight and increased disease scores were back to control values by day 13 post-challenge. BALF protein concentrations, however, remained significantly higher during the observation period (two weeks) of the study. Blood glucose concentration in the low vaccine dose (2.5 μg) group increased to significantly higher and in the high vaccine dose (40 μg) group remained significantly higher at the end of observation (Figure 4). 

RTA1-33/44-198 is not the only recombinant immunogen that provides a less than complete protection against the direct effects of the toxin on the airways. Thus, a transient weight loss and diminished lung function was observed after 10 × LD50 aerosol ricin challenge in mice vaccinated with different doses of RiVax [7]. Also, transient BALF protein concentration increases were observed after 3 × LD50 intra-tracheal ricin challenge in rats vaccinated with recombinant RTA [23]. These results underscore the difficulty of preventing direct cytotoxic effects at the portal of toxin entry and the importance of developing the ricin vaccine as part of an ensemble of physical and medical prophylaxis, as well as therapeutics.

Ricin intoxication was associated with a decrease in blood glucose concentration. It was surprising, therefore, to observe that challenges were associated with significant blood glucose concentration increases in some circumstances. Thus, such an increase occurred when mice were vaccinated with 40 μg doses of RTA1-33/44-198 and challenged with ricin (Figure 4). It was also observed when mice were vaccinated with 10 μg RTA1-33/44-198 and challenged with the same immunogen in place of ricin (Table 2). It also occurred with three of the VLP mutants described in this study. The blood glucose concentrations in the animals vaccinated with V76I, V76M and V76M/Y80A immunogens were significantly higher two days after ricin challenge than the values in the animals vaccinated with RTA1-33/44-198 (Table 4). When the blood glucose concentrations were compared with those in sham challenged animals, the values in the animals vaccinated with V76I were significantly higher, while the glucose concentrations in the animals vaccinated with the other two vaccines were comparable to the sham vaccinated control. The mechanism of this increase is unknown but it may be related to immune response because only the mice that were vaccinated with RTA1-33/44-198 and challenged with the same immunogen (in place of ricin) showed significant blood glucose concentration increase two days after challenge, whereas naïve mice challenged with the same immunogen did not (Table 2). Furthermore, the blood glucose concentration values in the immunized mice were significantly higher than in the naïve mice on both day 2 and day 13 after the vaccine immunogen challenge (Table 2). Additional studies are required to determine if these observations may be generalized to other animal challenge models or immunogens. 

None of the VLP mutants showed a major loss in immunogenicity as judged from their protective efficacy against the lethal effect of intranasal ricin challenge when compared to the RTA1-33/44-198 parent (Table 4). The only possible reduction in immunogenicity consistently observed was with the D75A mutant; mice vaccinated with D75A and challenged with ricin showed significantly elevated protein concentration in the BALF and significantly lower body weights two days after the challenge (Table 4). In contrast, neither a significant protein concentration increase in the BALF nor a significant body weight decrease was observed at the same time point when immunization occurred with a lower vaccine dose of the parent RTA1-33/44198 immunogen (Table 1).

Because the RTA-based proteins tested all undergo irreversible thermal denaturation that may be linked with aggregation, the observed unfolding curves and *T*_m_ values must be interpreted cautiously. Under identical experimental conditions, each VLP substitution reduced the apparent melting temperature compared with that of RTA1-33/44-198 (Figure 5). However, the measured *T*_m_ for D75N and V76I were within about 1 degree of RTA1-33/44-198 and, based on this superior quality and comparable immunogenicity, are considered the current lead candidates for further development. 

Our structure and *T*_m_ analysis demonstrated that the D75N mutation also could be successfully incorporated into the R48C/T77C or V49C/E99C disulfide bond variant. The D75N mutation was incorporated without compromising the experimental *T*_m_ or the structural integrity of known B-cell epitopes. Previous work showed that the disulfide bond variants of RTA1-33/44-198 retain immunoreactivity with toxin neutralizing antibodies *in vitro* [15] but further work is required to assess the efficacy of these novel immunogens with and without the VLP mutations *in vivo*. 

VLS was not observed in several pivotal animal trials employing comparatively high concentrations of RTA immunotoxins administered intravenously but it nevertheless caused serious and dose-limiting human toxicity in the clinic [11]. In our assessment of the literature, the VLS associated with RTA may be caused by the endogenous RIP activity of RTA and/or by the conserved VLP sequence identified and characterized extensively by Vitetta and colleagues [5,9,11]. Therefore, in developing a stabilized immunogen for ricin vaccines (RTA1-33/44-198) we intentionally removed the RIP catalytic activity from RTA by large-scale deletions. In this follow-on study, we show that the second potential cause of VLS can be removed from RTA1-33/44-198 by specific amino acid substitutions that result in little or no effect on the immunogen stability or its ability to protect mice from ricin exposure. 

## 4. Materials and Methods

### 4.1. Chemicals, Reagent Kits

Fast flow Q-Sepharose, SP-Sepharose and PD-10 columns were from GE Healthcare (Piscataway, NJ, USA). Phosphate buffered saline (PBS, NaCl 138 mM, KCl 2.7 mM, pH 7.4), 2,2,2-Tribromoethanol (tribromoethanol) and *tert*-amyl alcohol were from Sigma-Aldrich (St. Louis, MO, USA). Rehydrogel was purchased from Reheis (Berkeley Heights, NJ, USA). Ketaject (ketamine) and Xylaject (xylazine) were from Phoenix Pharmaceutical (St. Joseph, MO, USA). Ricin was purchased from Vector Laboratories (Burlingame, CA, USA). Micro BCA Protein Assay kit (Pierce, Rockford, IL, USA) and neutral buffered 10% (*v*/*v*) formaldehyde was purchased from Thermo Fischer Scientific (Rockford, IL, USA). QuikChange Multi Site-directed Mutagenesis kit was obtained from Stratagene (La Jolla, CA, USA). Wizard Plus SV Minipreps DNA Purification System was from Promega (Madison, WI, USA). BugBuster^®^ Protein Extraction Reagent was from Novagen (Darmstadt, Germany). Syringe filters (0.1 μm) were purchased from Millipore. *E. coli* BL-21(DE3) cells were purchased from Invitrogen (Carlsbad, CA, USA). Crystal Screen Cryo solution number 31 was obtained from Hampton Research (Aliso Viejo, CA, USA). 

### 4.2. Mutagenesis and Protein Purifications

Plasmid p188N [13] encoding RTA1-33/44-198 in pET24a(+) vector was used for site-directed mutagenesis with the QuikChange Multi Site-directed Mutagenesis kit. Oligonucleotide primers for the mutagenesis were synthesized and the mutations were verified with nucleotide sequence determination of both strands of purified plasmid DNAs. Parent and mutant plasmids were introduced into *Escherichia coli* BL21(DE3) for protein expression and proteins were purified as described [13] with minor modification (*i.e.*, the Mono-Q column was replaced with a fast flow Q-Sepharose column and the Mono-S column was replaced with a fast flow SP-Sepharose column). The codon-optimized DNA sequence and a gel of the purified proteins are shown in Figure S2. 

### 4.3. Circular Dichroism

Circular dichroism (CD) on protein solutions at 0.2 mg/mL in PBS was determined at 222 nm with a Jasco-815 CD spectropolarimeter (Easton, MD, USA). Thermal melt data were collected on triplicate samples of the proteins between 10 and 80 °C at a rate of 2 °C/min. The temperature at which 50% of the ellipticity is lost, the melting temperature (*T*_m_), was calculated using a four parameter fit.

### 4.4. Mice and Vaccinations

Fourteen to sixteen week old (for ricin LD50 determinations) and 6 to 8 week old (for immunization studies) female BALB/c mice were obtained from The Jackson Laboratory (Bar Harbor, ME, USA). Mice were treated in accordance with the guidelines for animal experimentation of the Walter Reed Army Institute of Research. Mice were immunized intramuscularly (i.m.) with 2.5 μg, 10 μg, or 40 μg of RTA1-33/44-198 or its mutant derivatives (dissolved in PBS) mixed with equal volumes of 2 mg/mL Rehydrogel (dissolved in 0.9% (*w*/*v*) NaCl) adjuvant. The injections were administered in the hind thigh in 100 μL volumes, alternating the left and the right sides during booster immunizations. Booster immunizations with the same vaccines and administration route were carried out two and four weeks after the priming immunization.

### 4.5. Intranasal Ricin Challenge

Challenges of mice were invariably carried out at a mouse age of 14–16 weeks (four weeks after the last booster immunization if vaccinated animals were used). Mice were anesthetized by i.m. injection of 30 μL of saline containing 600 μg/mL KetaJect and 180 μg/mL XylaJect in the hind thigh. The animals were turned on their backs and ricin in 50 μL volume was placed drop-wise on the nostrils for inhalation. Before use, ricin was dialyzed against PBS, filtered through a 0.2 μm sterile filter, its concentration determined with UV spectrophotometry using an extinction coefficient value of ε^1%^_cm_ = 11.8 at 280 nm [25]. The same batch of ricin was used throughout the studies. Control animals were challenged only with PBS under the same conditions (sham Challenge). In some of the experiments (as described in the text), ricin was replaced with vaccine immunogen. For these purposes, the protein concentration of the immunogen was determined with the Micro BCA Protein Assay Kit.

### 4.6. Survival, Body Weight and Disease Sign Observations, Blood Glucose and Bronchoalveolar Lavage Fluid Protein Concentration Determinations, Lung Histopathology

Immediately before challenge and throughout a 13-day observation period following challenge, body weights of mice were determined. Disease signs were also observed daily and scores were assigned according to the following scheme: 0, normal; 1, ruffled fur, normal activity; 2, ruffled fur, decreased activity; 3, hunchback posture, decreased activity; 4, dead. Mice were observed for survival twice daily. Blood glucose and bronchoalveolar lavage fluid (BALF) protein concentrations were determined in mice sacrificed either on day 2 post-challenge, or at the end of the observation period. After anesthetizing mice with intraperitoneal (i.p.) injection of 500 μL tribromoethanol-*tert*-amyl alcohol mixture (10 g 2,2,2-tribromoethanol dissolved in 10 mL *tert*-amyl alcohol and freshly diluted to 1:50 in PBS), the ribcage was opened to access the heart and lungs. After an incision into the heart, mice were let to bleed into the thoracic space. Blood glucose concentrations were measured immediately after making the incision in the heart before noticeable blood clotting using the handheld OneTouch Ultra blood glucose meter (Lifescan, Milpitas, CA, USA). After completely removing the blood from the thoracic space, a catheter was inserted into the trachea of the mice and the lungs were washed twice with 0.8 mL ice-cold PBS. The collected BALF was centrifuged at 5000 × *g* for 5 min at 4 °C to remove cells. The supernatant was used for protein concentration determination using the Micro BCA Protein Assay Kit. Lung histopathology was performed on some mice sacrificed on day 2 post-challenge. After anesthetizing mice with tribromoethanol-*tert*-amyl alcohol mixture as above, lungs were inflated with 10% (*v*/*v*) neutral buffered formaldehyde, removed, embedded and sectioned. Slides were prepared, stained with hematoxylin and eosin and evaluated for pathological changes with light microscopy.

### 4.7. Statistical Analysis

Differences between groups were analyzed for significance with an unpaired *t*-test. In cases where the data did not follow a normal distribution, non-parametric Mann-Whitney rank sum test was used to determine the significance of differences. Kaplan-Meyer analysis with Gehan-Breslow method followed by Holm-Sidak *post hoc* test for pairwise comparison of the datasets was used to determine time to death (TTD), time to body weight gain (TTWG) and time to disease-score drop (TTDD) values and the significance of their differences. The TTD was defined as the time between challenge (day 0) and death. For TTWG, the time elapsed from day 0 till the time the time for the first weight gain that was not followed by weight loss was used in the evaluations. In all comparisons a *p* < 0.05 was considered to be significant. The analyses were made by the appropriate suites of SigmaStat 3.5 (Systat Software, Richmond, CA, USA). Probit analysis was used to determine lethal dose 50 (LD50) using the BioStat statistical analysis program (AnalystSoft, Vancouver, Canada).

### 4.8. Crystallization of RTA1-33/44-198 R48C/T77C/D75N Disulfide Variant

RTA 1-33/44-198 D75N/R48C/T77C was purified using the BugBuster^®^ protocol described in Compton *et al* [15]. For crystallization, the protein was further purified using a G-200 Superdex column equilibrated with 50 mM MES pH 6.4 and 200 mM NaCl. Crystals were grown at 17 °C by hanging-drop in Hampton Crystal Screen Cryo solution # 31 (0.17 M Ammonium Sulfate, 25.5% PEG 4000, 15% glycerol) using a 1:1 ratio of protein to drop solution. Microseeding was required to obtain oval and half oval shaped sheet crystals, which were cryoprotected with Paratone-*N* prior to freezing. 

Diffraction data were collected with a Bruker Micro-STAR rotating anode equipped with Helios optics and a Bruker Platinum135 CCD area detector. Initial phases were calculated from PDB 3LC9 [15]. The structure was solved by molecular replacement using Amore [26]. Simulated annealing was done with CNS [27]. The model was refined with Refmac 5 [28] and model building and solvent addition was carried out in Coot [29].

## Supplementary Files

Supplementary File 1 Supplementary Information (DOC, 1942 KB)
