# Supplementary material for: Disruption of the Putative Vascular Leak Peptide Sequence in the Stabilized Ricin Vaccine Candidate RTA1-33/44-198
Source: Toxins (Basel). 2013 Jan 30;5(2):224–48. doi: 10.3390/toxins5020224 (PMC3640533; doi:10.3390/toxins5020224)
Supplement: Supplementary File 1 — Supplementary Information (DOC, 1942 KB) [file toxins-05-00224-s001.doc]

Supplementary Information

**Figure S1.** Comparison of X-ray crystal structure for (**A**) RTA bound to a transition state mimic (cyan) (PDB 3HIO) determined by Ho and colleagues [30] with that of
(**B**) RTA1-33/44-198 R48C/T77C structure (PDB 3LC9); (**C**) Overlay of the two structures. From the overlay it is apparent that a large portion of the substrate binding site is removed by the truncation of residues 34–43 (red) and 199-267 (light blue). No enzymatic activity has been observed for the RTA1-33/44-198 variant [31]. Three residues important to catalysis, Tyr-80, Asp-96 and Glu-177 are shown in yellow; (**D**) Enlarged views of the immunogens show how the VLP sites (orange) are exposed differently due to the *C*-terminal and 33/44-loop truncations. PDB 3LC9 is shown on the left and PDB 3HIO on the right; (**E**) Surface representations of 3LC9 and 3HIO; (**F**) Comparison of RTA (PDB 3HIO), 1-33/44-198 R48C/T77C (PDB 3LC9) with 1-33/44-198 R48C/T77C/D75N (PDB 4IMV). A salt bridge between Arg-56 and Asp-75 is lost in the D75N variant.

**A B C**


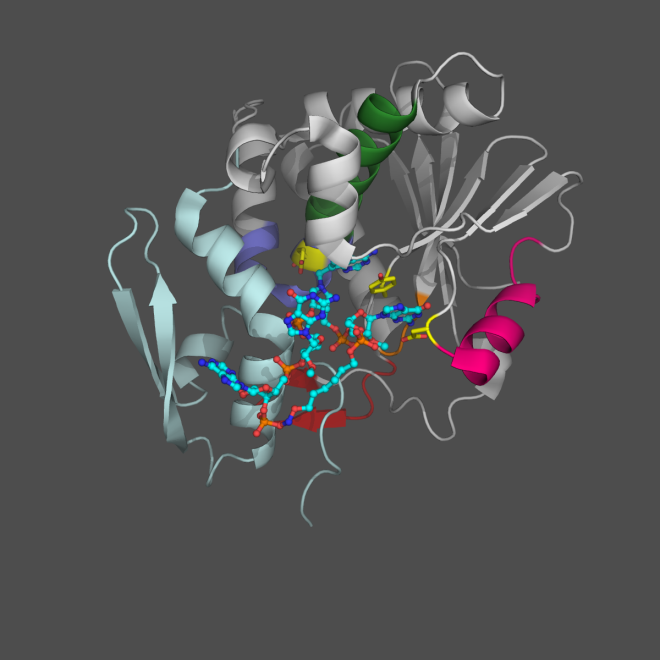

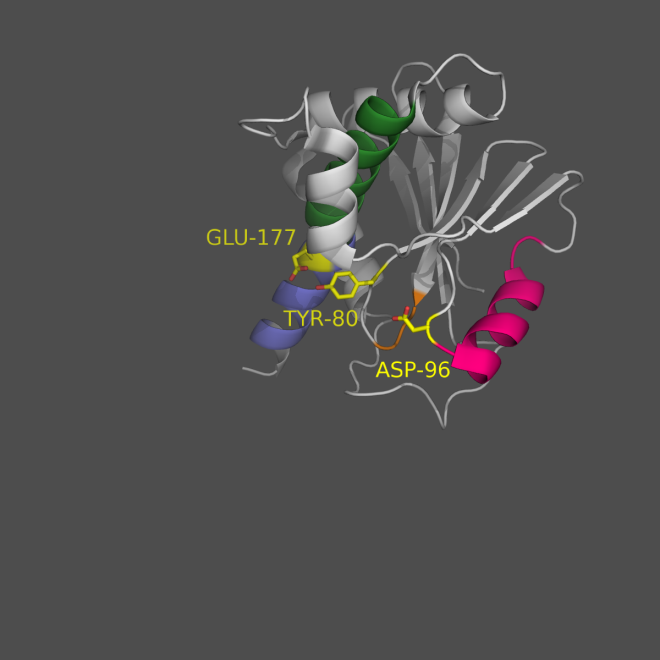

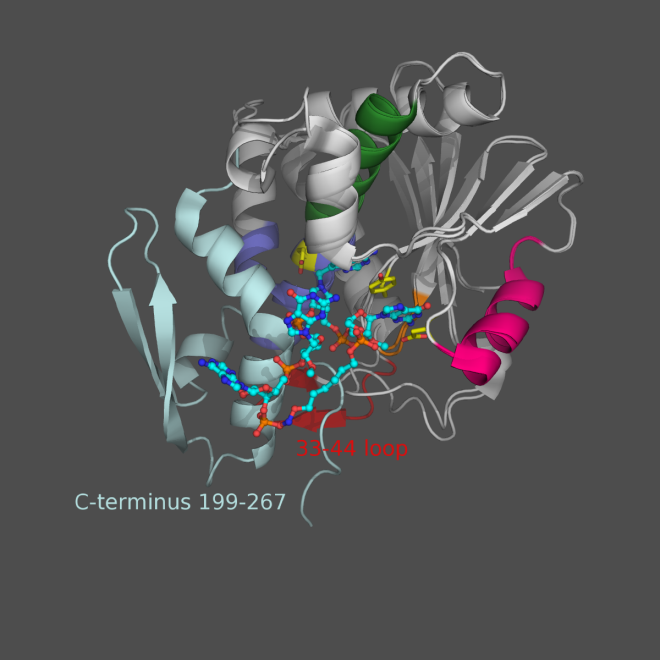


**D**


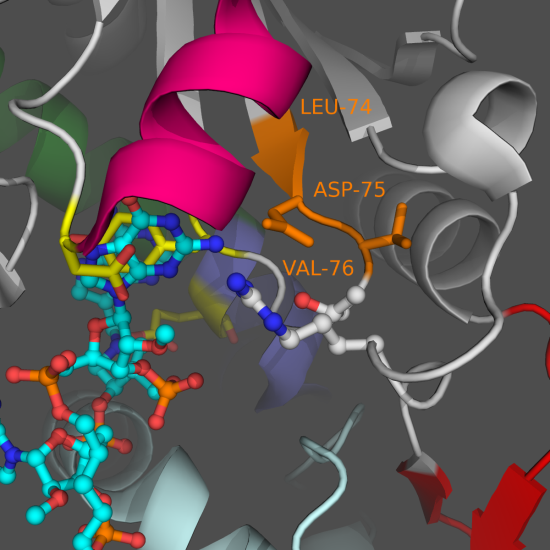

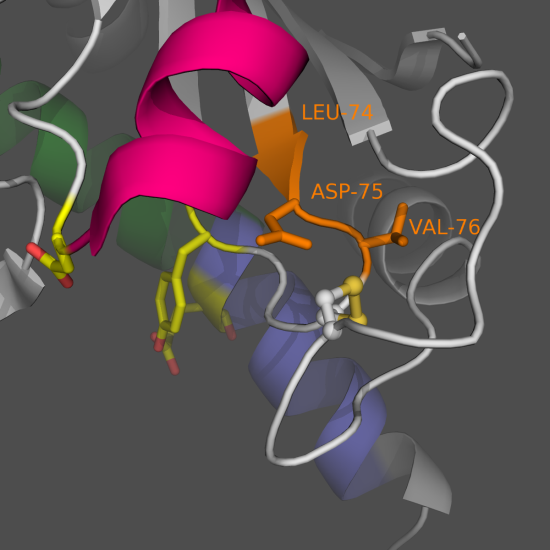


**E**


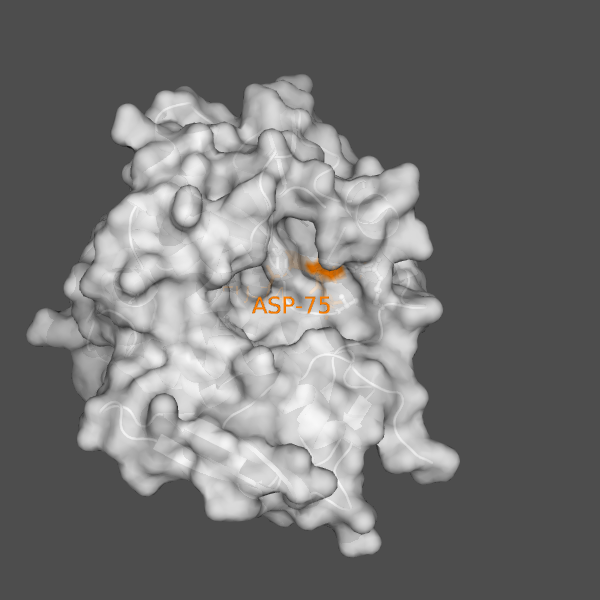

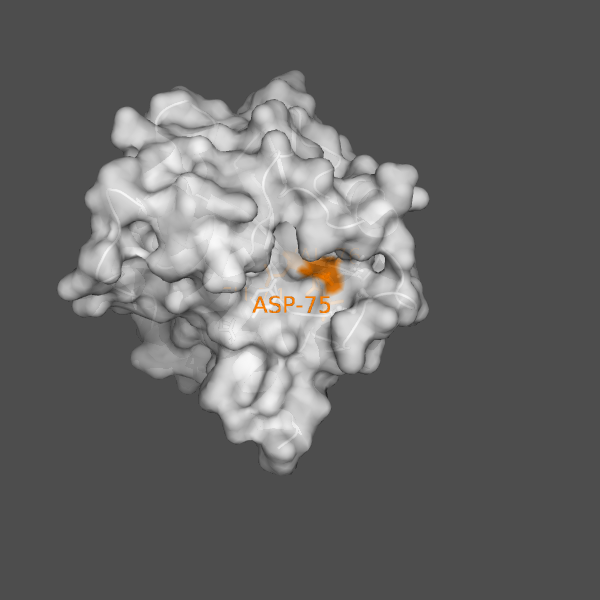


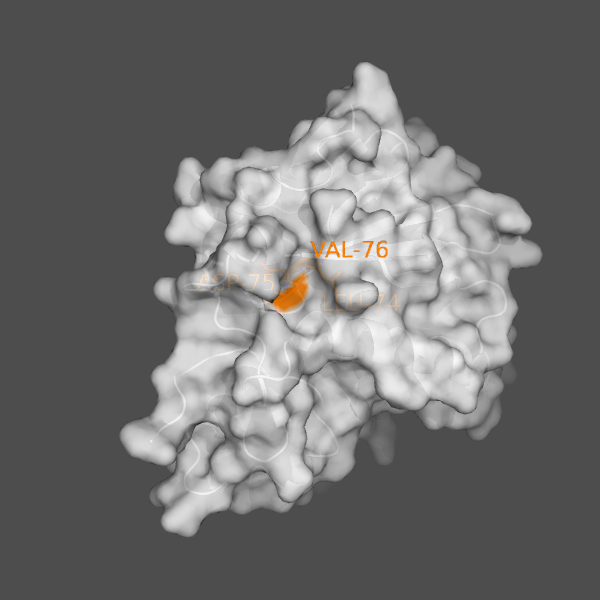

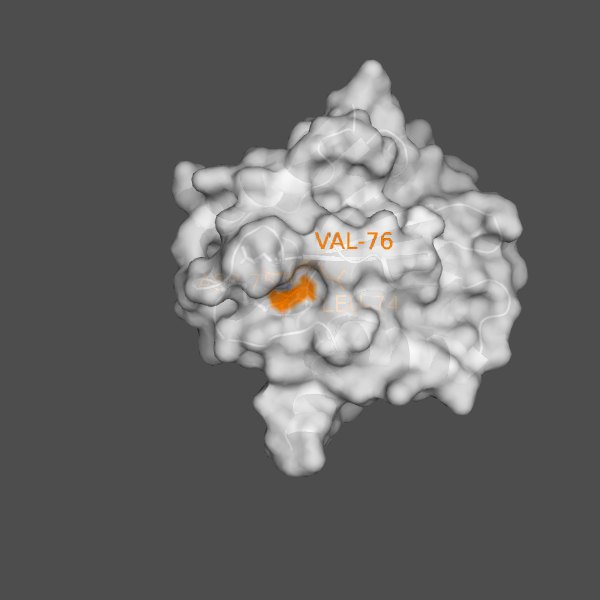


**F**

**Figure S2**. (**A**) The codon-optimized DNA sequence of the RTA1-33/44-198 construct utilized in this work; (**B**) The protein was expressed and purified from *E. coli* BL-21(DE3) as described. A gel of the purified recombinant proteins shows that the purified protein runs as a single band and is >95% pure (5 μg protein loaded per lane). Lane 1 contains RTA from *Ricinus communis* (Vector Labs, Inc.), Lane 2 contains recombinant RTA, Lane 3 contains RTA1-33/44-198, Lane 4 contains RTA1-33/44-198 R48C/T77C and Lane 5 contains RTA1-33/44-198 R48C/T77C/D75N.

**A**

ggataacaattcccctctagaaataattttgtttaactttaagaaggagatatacat**atg**

G - Q F P S R N N F V - L - E G D I H **M**

atcttcccgaaacagtacccgatcatcaacttcaccaccgcaggtgcaaccgttcagtct

I F P K Q Y P I I N F T T A G A T V Q S

tacaccaacttcatccgtgcagttcgtggtcgcctgaccgttctgccgaaccgtgttggt

Y T N F I R A V R G R L T V L P N R V G

ctgccgatcaaccagcgtttcatcctggtagaactgtctaaccacgcagaactgtctgtt

L P I N Q R F I L V E L S N H A E L S V

accctggcactggacgttaccaacgcgtacgtagtgggctaccgtgcgggtaactctgca

T L A L D V T N A Y V V G Y R A G N S A

tacttcttccacccagacaaccaggaggacgcagaagcaatcacccacctgttcaccgac

Y F F H P D N Q E D A E A I T H L F T D

gttcagaaccgttacaccttcgcgttcggtggcaactacgatcgtctggaacagctggca

V Q N R Y T F A F G G N Y D R L E Q L A

ggtaacctgcgtgagaacatcgaactgggtaacggtccgctggaagaggcgatctctgcg

G N L R E N I E L G N G P L E E A I S A

ctgtactactattctaccggtggtacccagctgccgaccctggcgcgttctttcatcatc

L Y Y Y S T G G T Q L P T L A R S F I I

tgcatccagatgatctctgaagcggcacgtttccagtacatcgaaggtgaaatgcgtacc

C I Q M I S E A A R F Q Y I E G E M R T

cgtatccgttacaaccgtcgttcttaggaattcgagctccgtcgacaagcttgcgccaca

R I R Y N R R S - E F E L R R Q A C A T

ctcgagcaccaccaccaccaccactgagatccggctgctaacaaagcccgaaagaagcta

L E H H H H H H - D P A A N K A R K K L

**B**

© 2013 by the authors; licensee MDPI, Basel, Switzerland. This article is an open access article distributed under the terms and conditions of the Creative Commons Attribution license (http://creativecommons.org/licenses/by/3.0/).
